# Impact of a Total Worker Health^®^ Mentoring Program in a Correctional Workforce

**DOI:** 10.3390/ijerph18168436

**Published:** 2021-08-10

**Authors:** Rajashree Kotejoshyer, Declan O. Gilmer, Sara Namazi, Dana Farr, Robert A. Henning, Martin Cherniack

**Affiliations:** 1 Department of Occupational and Environmental Medicine, Farmington, University of Connecticut Health Center, Farmington, CT 06030, USA; danafarr95@gmail.com (D.F.); cherniack@uchc.edu (M.C.); 2 Department of Psychological Sciences, University of Connecticut, Storrs, CT 06269, USA; declan.gilmer@uconn.edu (D.O.G.); robert.henning@uconn.edu (R.A.H.); 3 Department of Health Sciences, Springfield College, Springfield, MA 01109, USA; snamazi@springfieldcollege.edu

**Keywords:** corrections, health mentoring, healthy workforce participatory program, total worker health, peer mentoring, participatory action research

## Abstract

(1) Background: Correctional Officers show signs of adverse health early in their careers. We evaluated the impact of a one-year peer health mentoring program for new officers based on a Total Worker Health^®^ approach; (2) Methods: Cadets (*n* = 269) were randomly assigned to a mentored or control group. Cadets in this mixed methods design completed physical assessments, and surveys at three time points to assess demographics, health, mentoring, and workplace variables. Physical testing included several health markers. Surveys and physical data were analyzed as repeated measures. Regression analyses were used to analyze the relationship between mentoring characteristics and outcomes. A semi-structured interview of mentors was analyzed qualitatively. (3) Results: Higher mentoring frequency was associated with lower burnout. Health behaviors and outcomes declined over time in all groups, but mentees displayed slower decline for body mass index (BMI) and hypertension compared to controls. (4) Conclusions: A continuous peer health mentoring program seemed protective to new officers in reducing burnout and also declines in BMI and hypertension. Short-term physical health markers in younger officers may not be an index of psycho-social effects. A participatory design approach is recommended for a long-term health mentoring program to be both effective and sustainable.

## 1. Introduction

The risk to hazardous duty and law enforcement personnel (e.g., correctional officers (COs) and police officers) of developing serious chronic health conditions, such as cardiovascular disease, high blood pressure, and metabolic syndrome has been well-documented [1,2]. COs have the highest number of cases of non-fatal occupational injuries of all State employees [3]. In addition, COs are often exposed to mental health stressors at work and face high risk for anxiety, depression, and post-traumatic disorders [4,5]. Work stress and burnout in COs are linked to adverse health behaviors such as smoking, alcohol use, poor eating habits, and less physical exercise [1,4,5,6,7,8]. Job stress in COs is associated with substance abuse, work family conflict, suicide, and shortened life span [9,10,11].

Most COs begin their careers relatively early in adult life and retire younger than the average working population, which is related to the 20–25 year threshold for full pension benefits. New COs are required to pass a fitness test to demonstrate their physical health while entering employment, but their physical health, including musculoskeletal health, tends to quickly deteriorate within 24 months and stagnate in a pre-morbid state [11]. Increased obesity among COs during their early work tenure has also been reported, with mean body mass index (BMI) and body fat percentage increasing in the first three years of their employment [12]. These decrements in health indicators are concerning, as they may be linked to early-life chronic health problems, as well as chronic health problems in later adult working life, and to premature mortality.

Because of the early onset of health decline, it is critical to educate new COs about health risks associated with their job tasks during job orientation. Peer mentoring has been advocated as one way to help new COs adjust to their roles [13], and also may be a useful avenue specifically for health and well-being interventions at work. Traditional mentoring in the workplace is based on the constructs of Bandura’s social learning theory, as individuals learn from hierarchically senior members (mentors) in the organization [14,15,16]. Mentoring theory suggests benefitting individuals’ career progression as well as psychosocial aspects at work [15,16]. Peer mentoring refers to “relationships among individuals who are at a comparable organizational level in terms of pay, status, and job responsibilities” which have been found to have similar benefits as traditional mentoring with hierarchy [15,17]. Apart from corrections, “mentorship programs aid in mentees’ development of professional identity and competence and provide mentors a sense of generativity and purpose” [15,18,19,20]. Peer mentoring has been routinely instituted in many fields, but is new for the occupational health field [21].

Peer mentoring can have positive effects on organizational as well as individual outcomes. Mentoring may be helpful in reducing the effects of occupational stress. Specifically, occupational stressors are linked with reduced organizational commitment among COs [22], and organizational commitment has a well-supported negative relationship with turnover intentions [13,23,24,25]. A recent study suggests that early career CO peer mentoring could improve organizational commitment and reduce future turnover intentions [13]. It is important to note that mentoring programs are not monolithic, therefore, the quality of a mentoring relationship varies. We argue that the quality of mentoring may positively influence organizational outcomes. Peer mentoring programs can potentially be tailored to benefit individual health and organizational outcomes. From an analytic perspective, peer mentoring also provides a tripartite format for measuring outcome effectiveness (i.e., program: mentor: mentee) in contrast to bivariate associations (i.e., mentor: mentee).

The main aim of this study was to evaluate the impact of a peer health mentoring program (HMP) for new COs that involved peer mentors and was based on Total Worker Health^®^ (TWH) principles [26]. Principles of participatory action research (PAR) were applied to this peer HMP by involving experienced COs, union leaders, and representatives from management in program design, development and administration both for purposes of benefiting health and achieving program sustainability [27]. In general, we hypothesized that mentored cadets would demonstrate better health and perceived working conditions compared to their non-mentored counterparts across time points.

**Hypothesis** **1.** *Mentored cadets will display better physical health (body mass index, body fat percentage, blood pressure) across time points relative to control groups.*

**Hypothesis** **2.** *Mentored cadets will have better self-reported health/health behaviors across time points relative to control groups.*

**Hypothesis** **3.** *Mentored cadets will have better self-assessed working conditions across time points than control groups.*

A previous study in this population recognized a significant and long-lasting deterioration in health status in the first 2–5 years of employment [12]. Accordingly, we elected to conduct a longitudinal surveillance to evaluate health outcomes in the current study.

## 2. Materials and Methods 

The peer HMP in corrections was a collaborative effort of investigators from the Health Improvement Through Employee Control 2 (HITEC II) program and the Connecticut Department of Correction (DOC) [12]. HITEC II is a research program in the Center for the Promotion of Health in the New England Workplace (CPH-NEW) funded by The National Institute for Occupational Safety and Health (NIOSH) Total Worker Health Program. The study was approved by the Connecticut DOC and the University of Connecticut Institutional Review Board (IRB), protocol # IE-13-033S-2.

### 2.1. Study Design

A mixed methods study with a convergent parallel design was employed to evaluate the impact of the HMP based on TWH principles. A subset of new officer cadets assigned to mentors from the HMP were compared with a subset of new officer cadets that received conventional on-the-job training (OJT; Control group) using quantitative data. Additionally, qualitative data were extracted from semi-structured interviews of peer mentors to assess mentorship quality and emphasized CO’s individual experiences and perspectives rather than their representativeness of a distinct occupational group. The qualitative analysis was conducted using a phenomenological approach to understand the barriers to and facilitators of HMP success. This triangulation of the mentee quantitative data with the mentor qualitative data can be used to inform an in-depth understanding of the study design and impact of the program [28,29].

The key features of the peer HMP were (1) the labor-management participation in its design and implementation, and (2) line-level peer mentoring by COs guided by TWH principles.

A Study Wide Steering Committee (SWSC) provided oversight to HITEC II and also the HMP. The SWSC consisted of members of DOC management (e.g., deputy commissioner, director of human resources, research compliance officer), leaders at the facility level (wardens and their deputies), line-level COs and their bargaining unit, representatives of the supervisor’s bargaining unit, representative peer mentors, and research staff. The SWSC allocated compensated time for the HMP by covering release time for volunteer mentors training, peer mentoring, and evaluation, and also instructed supervisory staff in program accommodation. In addition, the presidents or designees of the three CO union bargaining units met separately to maintain the integrity of this CO-centric program.

The peer HMP approach to TWH featured individualized integration of work and health [30]. TWH is a central initiative from NIOSH and is defined as policies, programs, and practices that integrate protection from work-related safety and health hazards with promotion of injury and illness-prevention efforts to advance worker well-being. The TWH model adapted by our study center, CPH-NEW, involves integration of occupational safety and health with workplace health promotion using a core participatory approach which goes beyond a focus on individual behaviors in preventing chronic diseases [27]. TWH approaches incorporated into the HMP were distinct from the OJT with the addition of education on a set of health topics including behavioral strategies, such as exercising regularly despite a challenging shift schedule, eating healthy, managing stress, and addressing work-family issues. Thus, health mentoring considerably expanded upon conventional OJT for new officers by pairing them with specially trained peer mentors. Peer mentors were trained in an instructional class setting.

### 2.2. Materials

Participatory design focus groups were utilized to develop a training curriculum and reference handbook through a participatory design approach. A detailed description of the outcomes from these participatory design focus groups and the process of developing this HMP are beyond the scope of the current study and are described elsewhere in this special issue [31]. The handbook “Health Mentoring in the Correctional Workplace” was adapted from an existing career mentoring workbook from the Regionalization Project of 2002 by the Southern Region Division of the National Institute of Corrections Academy [32].

An additional focus group of supervisors and COs provided input on the best ways to train and recruit mentors and mentees. In order to recruit peer-mentors, and to encourage acceptance of unmentored comparison control groups, researchers presented the rationale for comparing mentored and non-mentored cadets, desired qualifications for mentorship, and mentors’ obligations and expected time commitment to all COs. Some experienced COs who volunteered to be mentors were recruited and recommended by their unions. Recruited peer-mentors were then trained in an instructional setting on health mentoring topics, and mentoring practices and procedures. Mentors received the handbook and a binder that included record keeping forms, an initial goal setting contract, and evaluation materials for mentee personal development and career goals at three months, six months, nine months, and at completion of the one-year HMP.

### 2.3. Participants and Procedures

Mentees were recruited by inviting new cadets into successive cadet classes at the DOC Training Academy and randomly assigning them to the HMP (intervention) or the conventional OJT (Control). In the project years, there were five classes matriculating at the Officers’ Training Academy in 2013 and 2014. Two classes were assigned to the Control, and three classes were assigned to the HMP. All cadets in each class were invited to participate in the study or to demur without consequence. Those choosing to participate were consented into the study. We distinguished study arms through separate training classes to minimize inter-group contamination.

Once peer-mentors were trained, research staff assigned mentees to a mentor as early as possible within their facility. Following mentor–mentee assignments, research study staff sent reminder emails and conducted follow-up visits to monitor the peer-mentors.

### 2.4. Instrumentation and Data Collection

All participant cadets (both HMP and Controls) completed a brief physical assessment and a comprehensive survey at three time points: Baseline at the academy while training as new cadets; Time 2 (T2) at the conclusion of the one-year peer HMP; and Time 3 (T3) at a later interval at the 5-year mark of employment.

The quality of the mentor–mentee relationship was assessed onsite with quarterly meetings between researchers and mentors with a brief semi-structured interview. The semi-structured interview was conducted with each peer-mentor by appointment to evaluate progress as well as barriers and facilitators to HMP processes. More specifically, the interview questions were structured to gain information on general mentoring progress, frequency and type of meetings with mentees (formal or informal), discussion topics with mentees, the creation of health goals with mentees, perception of mentoring, obstacles to mentoring success, and facilitators of peer health mentoring. Researchers collected a copy of progress notes if mentors provided them. Researchers made efforts to address any scheduling issues that mentors faced.

All evaluation results, once stripped of potential identifiers, were made available to the SWSC and participants in the mentoring program. This open programmatic review of findings was consistent with PAR principles and contributed to a process of continuous improvement.

The physical assessment measured height, weight, blood pressure, body mass index (BMI), and body fat percentage. Hypertension was categorized into five groups based on the systolic and diastolic measures; normal, elevated, stage 1, stage 2, and hypertension crisis [33]. BMI was calculated utilizing height and weight. Body fat percentage was calculated using the bioelectrical impedance method [34].

The HITEC All-Employee Survey assessed self-reported safety, physical and mental health, health behaviors, working conditions, and job factors. Job factors and working conditions consisted of items on physical and psychological job demands, decision latitude (decision authority, skill discretion), social support, emotional exhaustion (a component of burnout), work–family and family–work conflict, sense of coherence, and organizational health climate [34,35,36,37]. Details of these validated survey scales are described elsewhere [28]. The Center for Epidemiologic Studies depression scale (CESD-10) was used to assess depression symptoms. CESD-10 scores can range from 0 to 30, with a score of 10 or more indicating depressive symptoms [38,39,40]. Physical and Mental Health Composite Scores (PCS & MCS, 12 items) were computed using norming and calculation steps established by Ware and colleagues [40]. Scores can range from 0 to 100, where zero indicates lowest possible level of health and 100 indicates the highest level of health. The national norm for both physical and mental health composite scales are interpreted with a mean score of 50.0 and a standard deviation of 10.0 [41].

Health behaviors for nutrition (5 items; scale range 1–5), diet (5 items; scale range 1–4), and physical activity (4 items; scale range 1–6) were scored by calculating mean scores for all survey items within the topic. For these scales, higher scores indicate increased health-promoting behavior. Alcohol use was measured using two items: average number of alcoholic drinks consumed per week (1 = none or <1 drink to 5 = 21 + drinks) and number of times per week participants consumed 6 or more drinks in one sitting (i.e., binge drinking; 1 = never to 5 = daily or almost daily). Smoking was measured using one item with the following response options: “Have never smoked,” “Quit smoking 2 or more years ago,” “Smoke pipe or cigar only,” “Currently smoke less than 10 cigarettes per day,” and “Currently smoke 10 or more cigarettes per day.”

Workplace and job-related questions were eliminated at baseline as they were irrelevant prior to work assignment for the cadets but were included in the two follow-up surveys. All follow-up surveys and physical assessments were conducted at the mentee’s assigned facility, and all participants were provided their physical test results. Reliability measures (Cronbach’s alpha) of the previously validated scaled items utilized in this study sample were calculated and are reported in Section 3.1 of the Results section.

Mentees completed a quality of mentorship survey during their first follow-up evaluation that was used to assess the association between relationship quality, mentoring frequency, and outcomes at the first and second follow-ups.

### 2.5. Data Management and Analysis Plan

Participants were assigned random IDs and the de-identified dataset was used for the analysis. As noted, surveys and physical exams were voluntary and were approved by the University IRB. All data were confidential and were stored and secured within the university with access restricted to PI and research staff.

Data from the baseline All-Employee Survey, quality of mentorship survey of mentees, and physical health assessment were merged into one dataset by matching participant IDs. Follow ups at T2 and T3 surveys and physical assessments were linked to the baseline dataset by participant IDs.

All validated scale items were converted to scaled measures with scores for comparison analyses [34]. Survey descriptive statistics and cross-correlations for key subjective measures were also calculated.

As noted, a significant portion of participants in the HMP group were not assigned mentors or happened to terminate early; therefore, participants were grouped based on successful assignment and completion (see Table 1). There were potential mentees who were either unable to have or meet a mentor due to varying shifts or posts, or because they simply decided to no longer participate in the mentoring process. In order to avoid a dilution of effect, we assigned these participants to a “Non-compliant group” (*n* = 55), made up of individuals who were originally assigned into the mentoring group but never had a mentor for various reasons, which was separate from the “HMP group (*n* = 128)” and also from the Control (*n* = 86) group. Thus, comparisons were made on a grouped basis rather than intention to treat; the HMP group (mentored with assigned mentor), the Non-compliant group (no mentoring received from assigned mentor for HMP), and the Control group (on-the-job training).

One-way ANOVA and chi-square tests were conducted to establish differences between the groups at baseline on scored and categorical variables, respectively. Hypotheses 1 and 2 were tested with paired *t*-tests to evaluate health changes from physical assessments and self-reported health/health behaviors respectively among participants completing both baseline and T3 assessments in each study group. Hypotheses 1–3 were tested separately by conducting repeated measures ANOVA to determine differences in focal variables within and between HMP, Non-compliant, and Control groups across time points, while controlling for the focal variable at baseline. For Hypothesis 3 changes in working conditions were evaluated with paired *t*-tests from T2 to T3. Paired *t*-tests were appropriate for analysis of observed program impact on health and work changes, but the sample size reduced within each group across time points, thus limiting more differentiated analysis. Finally, additional regression analyses were conducted to determine if the quality of mentorship rating, or if the intensity of mentoring (meeting frequency), predicted subjective outcomes. The effect of mentoring was further evaluated by comparing mentees’ intention to turnover from follow-up surveys (T2 and T3). All analyses were performed in SPSS version 25.

All the semi-structured mentor interviews (*n* = 98) were transcribed and analyzed with NVivo qualitative software version 12.0. Commonly occurring themes discussed by all mentors were identified in this content analysis and analyzed by manual coding in the software. Verbatim quotes from the interviews are not presented in the results to protect confidentiality as per the institutional IRB approved protocol and participant consent.

## 3. Results

### 3.1. Health Mentoring Program Assessment

Among 406 eligible cadets in the HMP classes, 183 (45%) elected to participate; The Control group had a 42% (*n* = 170) participation rate. Participation was voluntary and participants were randomly assigned into mentoring or Control groups by class (rather than individually). Most mentors were assigned one mentee, but several mentors had either two or three mentees. The mean number of mentees per mentor was 1.14. Gender matching was done to the extent possible. Most of the mentees were male (76.4%), matching the high proportion of males in this working population. All cadets were assessed while at the Training Academy prior to facility assignment.

Our previous studies on COs found good reliability on survey items similar to the ones used here. This sample showed fair to good reliability for most measures. Reliability for various job factor measures were: physical job demands (Cronbach’s α = 0.74) psychological job demands (α = 0.44), decision latitude (α = 0.56), decision authority (α = 0.27), skill discretion (α = 0.49), social support (α = 0.71); supervisor support (α = 0.83), co-worker support (α = 0.57), burnout-emotional exhaustion (α = 0.59), work-family (α = 0.49), family-work conflict (α = 0.66), sense of coherence (α = 0.31), and organizational health climate (α = 0.85). Health behavior scales for nutrition (α = 0.77), diet (α = 0.88), and physical activity (α = 0.65) all demonstrated good reliability. The depressive symptoms score (α = 0.59) had somewhat lower reliability.

The baseline demographics of the three study cohort groups—HMP, Non-compliant, and Control—are reported in Table 1.

The HMP group was younger than the ‘Non-compliant’ and Control groups. For unknown reasons, the ‘Non-compliant’ group had a significantly higher level of education than the other two groups. The Control group had a slightly higher proportion of male cadets and lower education levels than the other groups (Table 1).

### 3.2. Baseline Mean Differences among Groups

Mean differences of physical health assessment, self-reported health, and health behaviors across the three mentor groups were evaluated at baseline. Sample size varied across all variables at baseline, as participation was voluntary and some participants chose to skip the survey and/or the physical assessment.

Overall, health status was not optimal at baseline, but it was similar across the three study groups (Table 2). Although there were no significant differences between the groups, the majority of participants had elevated blood pressure or hypertension stage 1 or 2 at baseline (83% in HMP group; 81% in Non-compliant group; and 77% in Control). Mean BMI and body fat percentage indicated all three groups in the overweight category at baseline based on standard weight status [41].

Chronic disorders diagnosis, defined as doctor-diagnosed heart attack, stroke, pre-diabetes, diabetes, hypertension, hyperlipidemia, asthma, chronic obstructive pulmonary disease (COPD), depression, skin cancer, other cancer, chronic recurrent back pain, or hearing loss, was low and similar across the groups at baseline. At baseline, more than one chronic disorder diagnosis was reported by 38% in the HMP group, 25% in the Non-compliant group, and 38% in the Control group, with no significant differences between groups (*p* > 0.05; Table 2).

Overall physical and mental health composite scales (PCS and MCS) at baseline were similar between groups and comparable to the general U.S. population scores (PCS = 50.2, MCS = 53.2; Table 2). Mean depression scores were low in all groups at baseline which indicated no clinically significant depressive symptoms (<10) (Table 2).

Baseline health behavior scores of nutrition, diet, and physical activity ranged from low to moderate and were similar across groups (Table 2). Of all participants at baseline, 17% reported currently smoking tobacco, 7.5% reported having more than 7 alcoholic drinks in a week, and 5.1% reported having 6 or more drinks in one sitting weekly. Chi-square tests revealed that these health behavior scores did not significantly vary between mentoring groups at baseline (*p* > 0.05).

Mean differences in self-assessed working conditions among study groups at baseline were not compared because cadets had just begun their jobs at DOC (Table 3).

### 3.3. Change in Health Measures, Health Behaviors, and Working Conditions within Each Mentor Group over Time

Because health changes emerge over time and because we chose to examine the longest possible interval to evaluate program effectiveness, we compared baseline to T3 to evaluate the health impact of the HMP. Overall, within-group differences for physical assessment variables and self-reported health measures on a paired *t*-test indicated declining health over time in all groups (Table 4).

The physical health quantitative measures of BMI and body fat percentage increased significantly in the HMP and Control groups from baseline to T3 follow-up. Hypertension increased from baseline to T3 significantly more in the Control group than in the HMP group (Table 4).

Self-assessed health data showed declining physical and mental health over time. Physical Health Composite scores declined from baseline to T3 significantly in the Control group (*p* < 0.05) but showed slight improvement in the HMP group (although not statistically significant, *p* > 0.05). Mental Health Composite scores declined significantly (*p* < 0.001) in each group from baseline to T3 (Table 4). Mean depression scores significantly increased in all groups over time from baseline to T3 (Table 4). Chronic disorder diagnoses increased over time, with more participants reporting doctor-diagnosed chronic disorders at T3 in all groups, but with significant increases in HMP and Non-compliant groups.

Healthy nutrition behavior and diet perception worsened significantly over time among all groups. The Control group worsened more from baseline to T3 than the other groups. Alcohol consumption (in terms of number of drinks per week) increased over time in all groups, but only increased significantly in the Non-compliant group between baseline and T3 (*t* (11) = 3.02, *p* = 0.012). Binge drinking did not change significantly within any of the three groups. Physical activity scores increased slightly over time in both the HMP and Control groups, but differences were not statistically significant (Table 4).

Changes in working conditions perceived by COs were assessed from T2 to T3 and were compared within each group with paired *t*-tests (Table 5). As noted previously, most perceptions of working conditions were not evaluated at baseline.

Overall perception of both physical and psychological job demands decreased significantly by T3 in all groups. Skill discretion increased in all three groups, although decision latitude decreased significantly in HMP and Control groups (Table 5).

Family-to-work conflict increased notably in both HMP and Control groups and decreased slightly in the Non-compliant group, but with no statistical significance. Work-to-family conflict significantly increased by T3 in the Non-compliant and Control groups but did not significantly increase in the HMP group (Table 5).

Although organizational health climate was perceived as slightly lower at T3 in all groups, the change was significant only in the Control group (*p* < 0.05; Table 5).

### 3.4. Comparison of the Three Groups over the Three Time Points with Repeated Health Measures and Working Conditions

No significant differences in health measures, health behaviors and working conditions were observed between the three study groups with repeated measures ANOVA. We have indicated only the statistically significant changes within groups over time in Table 6. The significant increase in BMI over time significantly differed among groups (Table 6).

Perceptions of physical demands, psychological demands, and decision latitude decreased in all groups from T2 to T3. Skill discretion improved slightly. As seen in Table 5, work-to-family and family-to-work conflict and health climate perception worsened in all three groups (Table 6).

Among health measures, depression, BMI, and hypertension increased significantly within groups over time (Table 6).

We did not see the hypothesized effect of peer health mentoring on health behaviors, as the nutrition and health importance scores declined in all groups over time (Table 6).

Additional regression analyses were conducted to determine if mentee-rated quality of mentorship and mentoring frequency at follow-up period T2 predicted the outcomes at T2 and T3 (scores for activity, depression, diet, exhaustion-burnout, physical composite scale, health climate, and sense of coherence) with age, gender, tenure, and shift at T3 as covariates.

Mentoring frequency was significant for reducing exhaustion-burnout at follow-up period T2 (β = −0.385, *p* < 0.01). It appeared that mentor quality and mentoring frequency did not predict any of the measured scores significantly at T3, several years after the mentoring relationship had ended.

### 3.5. Intention to Turnover among Groups

Response to the item, “I often think about quitting my job” was compared at T2 and T3 follow-ups. About 6% (HMP group), 11% (Non-compliant group), and 4.5% (Control group) (Bonferroni adjusted *p* > 0.05) at T2 responded “agree” or “strongly agree.” This proportion changed at T3 with about 25% (HMP group), 15.4% (Non-compliant group), and 30% (Control group) (Bonferroni adjusted *p* > 0.05) responding “agree” or “strongly agree.”

Reason for turnover intention was evaluated by the question “I am likely to leave this job in the next 2 years because I am dissatisfied” for both T2 and T3. About 5% (HMP group), 11% (Non-compliant group), and 1.5% (Control group) (Bonferroni adjusted *p* > 0.05) at T2 responded “agree” or “strongly agree.” This percentage changed at T3 with about 7.8% (HMP group), 7.7% (Non-compliant group), and 23.3% (Control group) (Bonferroni adjusted *p* > 0.05) responding “agree” or “strongly agree.”

### 3.6. Qualitative Analyses of Semi-Structured Quarterly Interviews with Mentors

Most mentors in the HMP group indicated that the content of their mentoring was mainly focused on work issues followed by stress management and healthy lifestyle, even though the latter two topics were the original focus of the HMP. Some mentors said that the reasons why they focused meetings on work issues were due to their lack of expertise and confidence in engaging in health mentoring. Mentors mostly met with their mentees informally. The binder provided to mentors to record meeting details was never used or maintained by most mentors; the reasons reported were that it was not short and simple to use.

A few mentors mentioned the mutual benefit of learning about themselves while working with their mentees. Some mentors mentioned becoming friends with mentees and meeting outside of work, or even receiving emotional support from their mentee. Some mentors were friends or acquaintances with their mentees through family or friends, and performed very well in these mentoring situations.

Mentors identified that meeting with mentees was easier when the mentees worked the same shift and in the same building/unit; when this was not the case, it was difficult for mentors to set up informal meetings with their mentees.

Mentors indicated the importance of supervisor support to meeting with mentees; in particular, supervisors providing relief time to meet with mentees was critical. Relief time provided by rover staff was found to be especially helpful. Only a limited number of mentors indicated that their supervisors and management were uncaring or being passive about the program. Other reasons for not meeting included disinterest in it even after receiving multiple reminders to meet and mentees’ lack of awareness about the long-term nature of the mentoring program. To improve the mentoring program, mentors suggested changes to mentee selection, with an option to choose their mentees, and also to have mentors formally introduced to mentees during their training classes at the academy.

**Figure 1:**
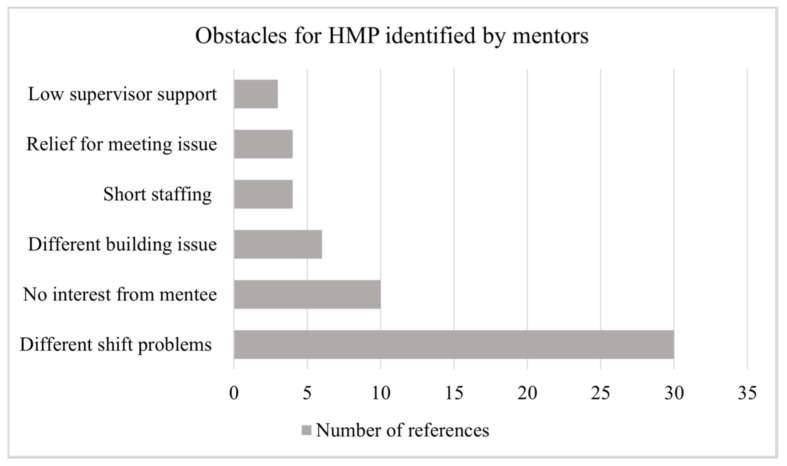
Obstacles for HMP.

Figure 1 demonstrates the number of times these obstacles for HMP were identified by mentors within these interviews.

## 4. Discussion

The main aim of this study was to evaluate the impact of the peer HMP on physical health measures and self-reported health perceptions, behaviors, and health outcomes. Overall, the differences between the HMP, Non-compliant, and Control groups in terms of physical health and psychosocial measures were relatively modest. Although hypertension worsened in all groups over time, it was lower in the mentoring group. BMI significantly worsened over time and significantly differed between groups, with the Control group having a greater increase in BMI than the other groups over time. Mentoring frequency was significant only for reducing exhaustion-burnout at T2 follow-up (β = −0.385, *p* < 0.01). We observed that unexplained variance within each group was large despite statistical and study controls.

Associations between meeting frequency with mentors and reduced exhaustion burnout among mentees in our study could be considered a positive impact of HMP, but additional research utilizing a larger mentee sample is warranted. A number of studies corroborate the need for further studies on exhaustion and burnout in this sector. Recent evidence from a TWH-based study among jail officers and deputies revealed higher post-traumatic stress disorder symptoms associated with job burnout among COs [42]. A related study reported high prevalence of depression among jail officers where job burnout was a significant predictor of depression symptoms after controlling for demographics and mental and physical health characteristics [43]. 

A mentoring study in Italy recently demonstrated that one year of formal mentoring promoted job adjustment and was protective against all facets of burnout (personal accomplishment, cynicism, and interpersonal strain) except for emotional exhaustion among new COs [44]. Our results from this longitudinal study show the protective role of peer mentoring in regard to emotional exhaustion as a component of burnout in new COs. Our results showing the positive effect of mentoring frequency and quality at the one-year conclusion of formal mentoring replicate the Italian results, although we did observe an extinction of protection by the fifth year during the follow-up evaluation of the longer-term effects of our HMP which had ended after one year. Significantly, new and improved mentoring program interventions are currently emerging in the DOC at this writing, thus substantiating the importance of a long-term and sustaining design.

Health behaviors such as smoking, alcohol use, and exercise worsened in all groups over the years in our study. Mentees exhibited slightly better health behaviors compared to controls, although not statistically significant. We consider this a positive observation and a protective effect of HMP that warrants further study. Shepherd et al. [7] reported that burnout among COs was associated with emotional demands and alcohol use for coping [7], suggesting that closer examination of emotional demands may be in order.

Our health measures were similar across all groups at baseline, and over time the differences between groups remained minimal. Our longitudinal findings replicate other studies on new COs that also indicate declining CO health with employment [1,12]. BMI and hypertension significantly increased within all groups in this study. Depression symptoms also increased significantly, with declining overall mental health composite scores of cadets. Physical composite scores decreased as well in all groups over time, but with no significant difference between groups. Cherniack et al. [12] reported similar health indicators in a cross-sectional study of established COs (*n* = 326). Although COs were relatively young in their study, with a mean age of 41 (7.2) years, the sample displayed numerous indicators of poor health. Eighty-five percent (85%) were considered overweight or obese (BMI of >25), 56% were either pre-hypertensive or hypertensive, and 31% screened positive for depressive disorders as measured by the CESD-10 [12]. COs in our study at T3 were younger than in previously reported studies [12], and depression with CESD-10 was screened positive in 30% of the HMP group and 30% of the Control group. The new cadets’ health at the start of their career was comparable to young COs in previous cross-sectional studies [1,12], thus confirming the robustness of the findings. Study results on self-reported work conditions varied. Physical and psychological demands were lower and skill discretion improved at T3 follow-up. Although it is tempting to conclude that adaptation leads to acceptance of work conditions, the results were not uniform, and turnover intentions increased. Evidence suggests that COs tend to have low decision latitude due to the hierarchical organizational structure within corrections and law enforcement workforces [5,45], and it seems likely this was also the case in the present sample.

COs’ intention to turnover was low at T2 (end of first year on the job) and had increased in all groups by the T3 follow-up. At the T3 follow-up, the proportion of individuals with the intention to leave their job was notably higher among the Control group as compared to the HMP group, although the difference was not statistically significant. Griffin et al. [13] examined turnover intent in COs over their career stages and reported a similar finding that turnover intent was lowest in COs in the first year of their job. They also reported that midcareer (5–9-year tenure) and younger COs who experienced a low level of organizational commitment expressed higher intent to leave the job [13]. Similarly, in our study, COs who were younger and had been working for five years had a higher intention to leave the job than their older age counterparts. We cannot ascertain whether mentoring could partially explain reduced turnover intention at T3 (at 5 years of employment) among the HMP group.

It is important to note that implementing the HMP required a significant investment of resources. Despite the high ranking of mentors and mentees of the program overall, a legitimate question is whether the results justify replication. While acknowledging that most health changes over time continued to be adverse, active mentoring lasted for only one year. The changes at the conclusion of the mentoring program did not appear to reflect better comparative health at T3.

The lack of more positive survey outcomes at T2 motivated closer examination of the underlying distribution of response patterns. We suspected the extreme working conditions COs face every day might contribute to response irregularities. For example, the high ratings of emotional labor in our analyses reflected that COs were likely managing their expressions and interactions out of fear of unfavorable inmate response, or the “tough” culture pressure from fellow officers, something described as the “John Wayne Syndrome” [6]. Problematic survey items that violated statistical assumptions (*n* = 21) were identified in the T1 and T2 datasets; twenty items had abnormal distributions (positively or negatively skewed, kurtosis, and heteroscedasticity issues) with one trending towards abnormal [46]. An effort was then made to address the root causes of item skewness before data collection at T3 [47]. Survey items identified as problematic were contextualized by subject matter experts on the research team so they would be more applicable to corrections. For example, we attempted to address potential ceiling effects in the original item, “How would you describe the quality of your sleep on a typical night?,” by removing “on a typical night” and adding “during the work week.” A preliminary examination of T3 data however, revealed only slight improvements in item skewness in adapted items [48]. This suggests that the value of using standardized survey items to evaluate targeted interventions like the HMP may be quite limited. Indeed, related work shows that participant-designed survey items can outperform standardized items because they incorporate individuals’ appraisals of their specific working conditions [48]. Therefore, we recommend that participatory design of survey items is utilized in any evaluation of peer HMPs in corrections [49]. We also see participatory design of survey items as central to the evaluation of TWH interventions in general. Program interventions are not likely to be “one and done” and will normally require continuous improvement over time through iterative design improvements that allow the program to respond to any new internal and external factors influencing work organization [50]. Findings from program evaluations with participants’ active role in designing are also more likely to be trusted and used to guide iterative design efforts. In fact, the present corrections organization’s new HMP reflects these iterative design efforts by now offering mentoring to officers throughout their tenure rather than only during their first year on the job.

The results indicate a decline in many measures of health over the first five years of employment in corrections, with mentoring during the first year not associated with the significant protective effect. There is no particular reason to believe that a one-year non-targeted program would be sustaining through the subsequent three decades of a working life, nor that measured effects would be large. The unanswered question is whether a longer-term continuous prevention program, or whether a program targeted to specific at-risk individuals, might have a more observable efficacy. Mentoring alone cannot control the working conditions or chronic physiological changes with work stress encountered by COs; however, a full-scale TWH approach to mentoring that combines individualized health interventions with organizational change could be helpful in combatting work stress and its health effects.

More than a decade before the HMP, the organization had experimented with a traditional mentoring program which was principally top-down in its implementation and was not sustainable. Its duration was short-term, its measures conventional, and it failed to secure any long-term health improvements. The peer HMP, created over a period of five years by an experienced and immersed study team, was largely a bottom-up creation which became a platform for further program development work. Its application was participatory, relying on the COs themselves to design and administer the interventions and to make corrections with the approach where needed. As such, it represented a significant progression of intervention techniques. Significantly, it reinforced in a very public way that COs are a high-risk population whose health status is a central problem in corrections. Addressing the implicit hazards of the job is tempered by the reality that COs are often a mobile group, and line officers require administrative support and experience to maintain their own peer-led program. The scheduling and staff coverage issues alone pose a challenge to a peer-based participatory program. Nielsen et al. [48] noted the complexity of evaluating participatory intervention programs due to their layered complexity [48]. Nielsen and Randall [51] also cited the lack of suitability of conventional survey methods in the assessment of this type of multi-featured intervention [51]. The HMP program relied on conventional design and assessment measures: (1) standard validated survey items, (2) a generic non-targeted approach to intervention, and (3) longitudinal introduction and refreshing deriving from the study team, not the participating workforce. Consequently, the HMP remained a blunt instrument, although, because of its participatory nature, it represented a significant evolution beyond the conventional administratively-provided mentoring intervention. As such, its next and ongoing iterations have incorporated our two most important observations: (1) that participatory mentoring developed and applied by the workforce itself is feasible, and (2) that the health and well-being needs of COs are potent and resistant to a generic approach.

Although well-being initiatives such as peer mentoring provide resources to COs adapting to their new environment, the prison system appears to need a culture change to reduce stress in the work environment and to reduce risks for associated chronic disease outcomes. Indeed, studies on COs’ well-being across multiple countries have indicated the value of a humane culture and lower violence and stress in prison systems [44,52]. A recent study on a correctional culture change intervention in the United States reported that a humane, health-promoting, and rehabilitation-focused prison system benefits the health and safety of correctional workforce as well as the incarcerated population [52]. Sustainability requires that CO enthusiasm and dedication to participatory health interventions should be met by institutional support and a recognition that improving a work culture is a continuous process. Our decade-long work with participatory health interventions within corrections has generated a culture change process marked by instances such as COs’ open discussions of mental health. Mental health interventions have and are currently being designed to improve workplace safety and officer health. Given the holistic nature of TWH interventions, it is imperative to evaluate effects on organizational support and culture along with physical and mental health outcomes of COs.

The declining health behaviors and health outcomes of new officers does not vitiate the importance of intervention. Correctional work culture differs by region and facility, thus requiring some customization. Overcoming the barriers to acceptance, including pessimism among COs, was a significant accomplishment of the HMP. A PAR mentor program facilitates adaptation of employees within a sector [27].

These lessons have been absorbed overall by the HITEC program as it continues with the evolution of the mentoring process. The ongoing descendant phase of interventions has incorporated the principal findings from the HMP. The first and most important was proof of the concept that trained personnel could conduct and improve a peer mentoring program. The second was that generic interventions were not fully suitable for the CO population with its variety of crises and issues, different states of urgency, and modest preparation of mentors. A third consideration was the language and construction of surveys, which were agnostic to the situational particularities of the CO workforce. In an earlier analysis on this same workforce, we had observed surprisingly blunted CO responses [34].

Because the resources invested were substantial and effects modest, unmodified duplication of the HMP seems unadvisable. However, there are several merits to the HMP and its approach and, as noted, there is active modification which builds on the process that can be expected to be self-correcting via continuous improvement. These merits include the high morale of participating mentors and mentees towards the HMP, the enthusiasm of COs and union leadership towards participatory action, and the willingness of upper management to support these grassroots efforts.

First, by involving COs in the development and continuation of the HMP, we were able to overcome widespread CO resistance and distrust towards preventive health and work programs. CO involvement was also effective in alleviating privacy concerns and COs’ general reluctance to share personal health information with fellow officers. Additionally, the labor-management group was able to dissolve formidable obstacles and maintain the participatory program, which is a considerable organizational process achievement. Furthermore, the participatory labor-management approach provides a platform for revision and continuous improvement of the program over time. Currently, the mentoring program has been adapted for peer involvement of all COs at the facility level regardless of job tenure. There is utilization of the training materials and ongoing training of engaged COs in mental health supports and interventions. While that process is not the subject of this manuscript, it may justify the degree of investment in a joint labor management program for peer health mentoring.

The emphasis on voluntary participation in a hierarchical environment also influenced self-selection and adherence. We had a small study cohort of 269 with unequal distribution of groups due to programmatic challenges, which may have diluted results. Efforts were made to align peer-mentor and mentee work shifts, but experienced potential mentors were first shift workers, whereas assignment to the second and third shifts was more common for new officers.

The complexity of testing three groups at three time points with repeated measures can lead to sampling bias and Type-1 error. To reduce bias statistically, we conducted tests for unequal variances and sphericity violations.

One of the inherent problems with a repeated-measures design is participant attrition over time and sample reduction. Although we had acquired relief time for participants, we acknowledge that the survey length and pressure to get back to post during follow-ups may have affected participation. Nonetheless, mentor recruitment efforts at the cadets’ destination facilities were found to be effective, with the number of volunteer mentors more than doubling in the second year of the program, and few established mentors dropping out over two years. As a programmatic issue, the recruitment process resulted in three groups rather than the planned two groups, because of the distinct status of what we termed ‘non-compliance,’ which reduced statistical power. This might have affected the lower Cronbach’s alpha of scales in our study compared to a similar population in corrections utilizing similar scaled measures [34].

Due to varying schedules and officers being transferred to other facilities, some mentor pairs were considered non-compliant. However, consistent with a PAR approach, programmatic adjustments were made over time, such as securing greater program facilitation by captain and lieutenant supervisors so that meetings between mentors and mentees working on the same shift could occur on a more regular basis. Our qualitative data indicate that it is also helpful to work in the same building/unit such that physical meetings are more feasible. Introducing mentees to mentors in a more formal way at the training academy was another suggested program improvement yielded by the qualitative data. Informal mentoring was adopted by many mentors in this study; future studies could explore the effectiveness of formal versus informal peer health mentoring of COs.

Finally, this was not, contextually, a negative study. Its lessons go beyond the conventional defaults applied to public health studies that no single study is definitive, or that longer duration and larger populations are necessarily merited. In the context of ongoing participatory interventions directed to organizational and cultural change, the outputs from the HMP program were highly positive and effective. Viewed contextually, a conventional and conservative study design produced non-enduring positive results that returned to the null over a more extended period of observation. Both the short duration effectiveness of the participatory program and the limitations of transience are being addressed through this process of cultural change in corrections.

## 5. Conclusions

This study presents these key contributions to the literature and applied work on correctional officer health:

1. A longitudinal study which tracked new correctional officer demographic variables, work conditions, subjective health, and physical health indicators over a period of 5 years.
2. Development of a peer health mentoring program using participatory action research principles in a correctional officer population which included a labor-management participatory design approach.
3. Programmatic approaches for workplace health promotion consistent with Total Worker Health principles.
4. Documentation of health risks specific to the correctional officer population.

Mentoring frequency was associated with reduced exhaustion-burnout among mentees. Slow decline in health measures of body mass index and hypertension among mentees can be considered a positive effect of mentoring. We did not observe any other direct impact of the mentoring program on health behaviors and health outcomes in this study. It is of utmost importance to address work and health conditions to improve COs’ declining physical and mental health for a healthy future of work in corrections. Although the impact was limited, this PAR approach that engaged COs and union leaders with the support of management was effective in developing and implementing this officer-led peer health mentoring program based on TWH principles. A health mentoring program can be expected to be more effective over the long term with a built-in participatory design approach for continuous improvement that empowers COs to advance the well-being of their workforce.

The barriers to successful health interventions in corrections can be formidable. However, this initial HMP provided a pathway towards establishing more effective and sustainable mentoring programs to improve the quality of work life and support improved health for COs. As noted, the HMP is part of an iterative continuous improvement process in the organization that can grow over time. CPH-NEW has developed a new toolkit to guide participatory development of HMPs that incorporates lessons learned from this HMP and subsequent adaptations of the program [49], with plans for updating this tool in the years ahead.

## Figures and Tables

**Table 1:** Demographics within study groups at baseline (*n* = 269).

		Health Mentoring Program (HMP) Group (*n* = 128)	Non-Compliant Group (*n* = 55)	Control Group (*n* = 86)
Mean Age (SD)		30.38 (5.76)	32.38 (7.19)	32.47 (7.22)
Gender (%)	Male	75.8%	74.5%	84.9%
	Female	24.2%	25.5%	15.1%
Education Level		Percent (*n*)	Percent (*n*)	Percent (*n*)
High school graduate or GED		16.4% (20)	14.9% (7)	22.7% (15)
Some college		41.0% (50)	25.5% (12)	34.8% (23)
College degree (2 or 4 year)		39.3% (48)	57.4% (27)	37.9% (25)
Graduate degree		3.3% (4)	2.1% (1)	4.5% (3)

**Table 2:** Comparison of physical health assessments, and self-assessed health outcomes among study groups in all 3 time points.

Physical Health Assessments		**Health Mentoring Program (HMP)** **Group** (***n* = 128**)**,** **Mean** (**SD**)			**Non-Compliant Group** (***n* = 55**)**,** **Mean** (**SD**)			**Control Group** (***n* = 86**)**,** **Mean** (**SD**)		
		Baseline	T2	T3	Baseline	T2	T3	Baseline	T2	T3
	Body fat %	24.76 (7.70)	25.32 (7.97)	28.19 (9.09)	24.58 (8.66)	26.63 (10.43)	30.67 (7.23)	22.85 (7.54)	24.62 (7.23)	26.19 (6.58)
	Body Mass Index (BMI)	28.95 (4.61)	29.91 (4.98)	30.37 (5.22)	28.73 (4.74)	31.12 (6.35)	32.03 (5.26)	29.09 (4.39)	30.46 (4.76)	30.98 (4.18)
	Hypertension score	2.68 (1.03)	3.10 (0.99)	2.95 (1.12)	2.68 (1.07)	3.03 (1.08)	2.50 (1.16)	2.64 (1.12)	2.97 (1.01)	3.06 (1.08)
Self-Assessed Health	Physical Health Composite Score (PCS)	51.36 (5.31)	50.46 (6.28)	50.50 (6.96)	52.11 (5.45)	52.31 (5.46)	51.74 (5.35)	51.36 (4.76)	51.85 (4.78)	48.32 (8.45)
	Mental Health Composite Score (MCS)	53.41 (5.80)	52.06 (6.79)	40.46 (7.50)	53.92 (4.69)	51.70 (7.86)	41.42 (5.57)	53.36 (6.49)	53.61 (6.05)	41.44 (7.03)
	Depression score	3.93 (1.52)	4.59 (2.16)	7.54 (4.28)	3.49 (1.47)	4.00 (1.75)	7.00 (3.94)	3.52 (1.88)	3.98 (1.84)	7.80 (7.14)
	Chronic disorder diagnosis score	0.55 (1.02)	0.39 (1.13)	1.46 (0.59)	0.38 (0.73)	0.30 (0.52)	1.57 (0.53)	0.65 (1.16)	0.49 (0.89)	1.83 (1.20)
Health Behaviors	Nutrition behavior score	2.84 (0.77)	2.62 (0.82)	2.27 (0.79)	2.71 (0.73)	2.56 (0.87)	2.43 (0.79)	2.99 (0.79)	2.84 (0.79)	2.35 (0.57)
	Diet score	3.03 (0.52)	2.95 (0.61)	2.56 (0.70)	3.02 (0.52)	2.93 (0.64)	2.75 (0.41)	3.07 (0.57)	3.08 (0.66)	2.44 (0.63)
	Activity	3.08 (0.81)	3.07 (0.93)	3.09 (1.05)	2.99 (0.87)	3.07 (0.89)	2.92 (0.95)	2.94 (0.80)	3.10 (0.93)	3.00 (1.15)
	Smoking (% have never smoked)	57.38%	56.57%	48.15%	64.58%	64.86%	53.85%	58.46%	59.09%	56.67%
	Alcohol Use (1 = none or <1 drink, 5 = ≥ 21 drinks/week)	1.50 (0.66)	1.63 (0.67)	1.76 (0.75)	1.65 (0.83)	1.65 (0.71)	2.08 (0.86)	1.65 (0.76)	1.70 (0.85)	2.10 (0.99)
	Binge drinking (≥6 drinks in one sitting; 1 = never, 5 = daily/ almost daily)	1.72 (0.87)	1.90 (0.95)	1.98 (0.96)	1.75 (0.93)	1.78 (1.02)	2.23 (1.09)	2.00 (0.88)	1.96 (0.99)	2.20 (1.13)

Note: One-way ANOVAs for scored variables and Chi-square tests for categorical variables showed no significant differences among study groups at baseline. Sample size (*n*) varies among variables due to missing responses.

**Table 3:** Comparison of self-assessed working conditions among study groups in all 3 time points.

Self- Assessed Working Conditions		**Health Mentoring Program** **Group** (***n* = 128**)**,** **Mean** (**SD**)			**Non-Compliant Group** (***n* = 55**)**,** **Mean** (**SD**)			**Control Group** (***n* = 86**)**,** **Mean** (**SD**)		
		Baseline	T2	T3	Baseline	T2	T3	Baseline	T2	T3
	Sense of coherence	3.84 (0.61)	3.83 (0.60)	3.82 (0.49)	3.98 (0.58)	3.78 (0.55)	3.50 (0.84)	3.89 (0.58)	3.98 (0.67)	3.82 (0.79)
	Exhaustion-burnout	---	3.73 (1.21)	4.15 (1.19)	---	3.52 (1.22)	3.67 (1.05)	---	3.43 (1.13)	4.20 (1.27)
	Health climate	5.67 (1.42)	4.40 (1.74)	4.19 (1.10)	5.75 (1.20)	3.93 (1.56)	4.36 (0.73)	5.54 (1.58)	4.41 (1.54)	4.02 (0.97)
	Family to work conflict	1.41 (0.47)	1.39 (0.43)	1.69 (0.52)	1.35 (0.45)	1.32 (0.43)	1.40 (0.52)	1.38 (0.51)	1.27 (0.37)	1.78 (0.61)
	Work to family conflict	1.64 (0.48)	1.79 (0.56)	1.92 (0.74)	1.61 (0.47)	1.66 (0.44)	2.00 (0.58)	1.66 (0.51)	1.66 (0.56)	2.00 (0.71)
	Decision authority	---	3.30 (0.44)	2.60 (0.50)	---	3.28 (0.35)	2.63 (0.51)	---	3.38 (0.35)	2.58 (0.63)
	Decision latitude	---	2.90 (0.28)	2.66 (0.46)	---	2.89 (0.22)	2.85 (0.44)	---	2.94 (0.25)	2.64 (0.60)
	Skill discretion	---	2.51 (0.28)	2.73 (0.63)	---	2.51 (0.29)	3.07 (0.41)	---	2.51 (0.33)	2.70 (0.71)
	Physical job demands	---	3.05 (0.46)	1.98 (0.50)	---	2.89 (0.38)	2.47 (0.55)	---	3.02 (0.42)	2.10 (0.52)
	Psychological job demands	---	3.23 (0.30)	2.43 (0.46)	---	3.09 (0.32)	2.41 (0.39)	---	3.24 (0.35)	2.30 (0.38)
	Social support	---	2.70 (0.33)	2.76 (0.62)	---	2.67 (0.25)	2.73 (0.53)	---	2.64 (0.34)	2.75 (0.70)

Note: One-way ANOVAs for scored variables showed no significant differences among study groups at baseline. Sample size (*n*) varies among variables due to missing responses.

**Table 4:** Change in physical health assessments and self-assessed health outcome mean scores from baseline to T3 within each study group.

Physical Health Assessments		**Health Mentoring Program (HMP) Group-Mean Difference T3-Baseline** (**SD**)**,** ***t*** (**df**)	**Non-Compliant Group-Mean Difference T3-Baseline** (**SD**)**,** ***t*** (**df**)	**Control Group-Mean Difference T3-Baseline** (**SD**)**,** ***t*** (**df**)
	Body Mass Index (BMI)	1.95 (2.82), *t* (50) = 4.93 ***	1.97 (3.04), *t* (12) = 2.35 *	2.03 (2.77), *t* (34) = 4.34 ***
	Body fat %	3.40 (5.63), *t* (46) = 4.14 ***	2.47 (4.02), *t* (10) = 2.04	3.45 (4.88), *t* (33) = 4.12 ***
	Hypertension score	0.24 (1.24), *t* (53) = 1.42	−0.14 (1.56), *t* (13) = −0.34	0.51 (1.31), *t* (34) = 2.32 *
Self-Assessed Health	Physical Health Composite Score (PCS)	0.05 (6.99), *t* (49) = 0.049	−0.29 (5.62), *t* (12) = −0.19	−3.87 (8.25), *t* (23) = −2.30 *
	Mental Health Composite Score (MCS)	−13.16 (7.71), *t* (49) = −12.07 ***	−11.57 (8.13), *t* (12) = −5.14 ***	−12.53 (7.38), *t* (23) = −8.31 ***
	Depression score	3.25 (3.50), *t* (51) = 6.69 ***	3.17 (4.76), *t* (11) = 2.30 *	4.27 (6.71), *t* (25) = 3.25 **
	Chronic disorder diagnosis score	0.74 (0.81), *t* (22) = 4.38 ***	0.85 (0.69), *t* (6) = 3.29 *	0.69 (2.36), *t* (15) = 1.17
Health Behaviors	Nutrition behavior score	−0.43 (0.82), *t* (52) = −3.80 ***	−0.38 (0.55), *t* (10) = −2.31 *	−0.58 (0.63), *t* (25) = −4.66 ***
	Diet score	−0.38 (0.71), *t* (51) = −3.85 ***	−0.22 (0.44), *t* (10) = −1.68	−0.64 (0.81), *t* (22) = −3.80 **
	Activity	0.26 (0.99), *t* (51) = 1.88	−0.31 (0.72), *t* (11) = −1.51	0.19 (1.01), *t* (25) = 0.97
	Alcohol Use (1 = none or < 1 drink, 5 = ≥ 21 drinks/week)	0.12 (0.78), *t* (51) = 1.06	0.58 (0.67), *t* (11) = 3.02 *	0.24 (0.83), *t* (24) = 1.45
	Binge drinking (≥6 drinks in one sitting; 1 = never, 5 = daily/almost daily)	0.10 (0.91), *t* (51) = 0.76	0.42 (1.16), *t* (11) = 1.24	0.08 (1.19), *t* (24) = 0.34

Significance levels for paired *t*-tests within each group: * Significant at *p* < 0.05; ** Significant at *p* < 0.01; *** Significant at *p* < 0.001.

**Table 5:** Changes in self-assessed working conditions means from T2 to T3 within each study group.

Self- Assessed Working Conditions		**Health Mentoring** **Program Group-** **Mean Difference** **T3–T2** (**SD**)**,** ***t*** (**df**)	**Non-Compliant Group-** **Mean Difference** **T3–T2** (**SD**)**,** ***t*** (**df**)	**Control Group-** **Mean Difference** **T3–T2** (**SD**)**,** ***t*** (**df**)
	Sense of coherence	0.03 (0.61), *t* (44) = 0.32	−0.37 (0.76), *t* (9) = −1.52	−0.27 (0.80), *t* (26) = −1.76
	Exhaustion-burnout	0.24 (1.17), *t* (44) = 1.40	0.07 (1.00), *t* (8) = 0.22	0.57 (1.56), *t* (26) = 1.92
	Health climate	−0.41 (1.75), *t* (43) = −1.55	0.11 (1.25), *t* (7) = 0.25	−0.46 (1.02), *t* (26) = −2.34 *
	Family to work conflict	0.34 (0.65), *t* (46) = 3.58 **	−0.15 (0.53), *t* (9) = −0.90	0.52 (0.69), *t* (26) = 3.93 **
	Work to family conflict	0.09 (0.63), *t* (45) = 0.93	0.40 (0.39), *t* (9) = 3.21 *	0.39 (0.76), *t* (26) = 2.65 *
	Decision authority	−0.67 (0.63), *t* (43) = −7.06 ***	−0.73 (0.80), *t* (9) = −2.91 *	−0.81 (0.68), *t* (26) = −6.27 ***
	Decision latitude	−0.22 (0.46), *t* (43) = −3.20**	−0.13 (0.53), *t* (9) = −0.79	−0.29 (0.53), *t* (26) = −2.82 **
	Skill discretion	0.22 (0.69), *t* (43) = 2.15 *	0.47 (0.49), *t* (9) = 3.01 *	0.24 (0.62), *t* (26) = 2.03
	Physical job demands	−1.08 (0.60), *t* (43) = −11.94 ***	−0.50 (0.79), *t* (8) = −1.90	−0.92 (0.67), *t* (26) = −7.16 ***
	Psychological job demands	−0.77 (0.50), *t* (43) = −10.15 ***	−0.75 (0.50), *t* (8) = −4.50 **	−0.82 (0.54), *t* (26) = −7.87 ***
	Social support	0.04 (0.75), *t* (43) = 0.40	0.08 (0.63), *t* (8) = 0.56	0.09 (0.74), *t* (26) = 0.65

Significance levels for paired *t*-tests within each group: * Significant at *p* < 0.05; ** Significant at *p* < 0.01; *** Significant at *p* < 0.001.

**Table 6:** Repeated measures comparison across groups for health and working conditions.

		Within Groups (Time * Study Group)		Between Groups
		Time	Time * Study group	Study group
		F-statistic ^a^ (df)	F-statistic (df)	F-statistic (df)
Physical Health Assessments (Baseline, T2, T3)	Body Mass Index (BMI) (*n* = 85)	41.10 (1) ***	5.23 (2) **	0.45 (2)
	Hypertension (*n* = 90)	5.07 (1) *	0.16 (2)	0.33 (2)
Self- Assessed Health (Baseline, T2, T3)	Depression (*n* = 73)	21.60 (1) ***	0.02 (2)	2.03 (2)
Health Behaviors (Baseline, T2, T3)	Nutrition score (*n* = 76)	14.07 (1) ***	0.69 (2)	1.29 (2)
	Health importance score (*n* = 75)	6.71 (1) *	0.24 (2)	3.07 (2)
Self-Assessed Working Conditions (T2, T3)	Physical demands (*n* = 80)	87.82 (1) ***	3.11 (2)	1.32 (2)
	Psychological demands (*n* = 80)	120.01 (1) ***	0.10 (2)	1.11 (2)
	Decision latitude (*n* = 81)	10.64 (1) **	0.38 (2)	0.82 (2)
	Skill discretion (*n* = 81)	12.98 (1) **	0.58 (2)	1.59 (2)
	Work to family conflict (*n* = 76)	8.43 (1) **	0.26 (2)	0.03 (2)
	Family to work conflict (*n* = 77)	4.35 (1) *	0.28 (2)	0.08 (2)
	Health climate (*n* = 71)	22.23 ^b^ (1.8) ***	0.30 (3.6)	0.36 (2)
	Sense of coherence (*n* = 75)	5.12 (1) *	2.72 (2)	0.94 (2)

^a^ F-statistic from repeated measures ANOVA for quadratic trend reported where linear trend was non-significant. ^b^ Greenhouse–Geisser values used because sphericity assumption was violated. Significance levels for F-statistic: * Significant at *p* < 0.05; ** Significant at *p* < 0.01; *** Significant at *p* < 0.001.

## Data Availability

Data presented in this study is available on request from the corresponding author. The data are not publicly available due to confidentiality concerns with consent agreement and organizational rules/policies with IRB and DOC.

## Abbreviations

CO	correctional officer
HMP	health mentoring program
HITEC	Health Improvement Through Employee Control
CPH-NEW	Center for the Promotion of Health in the New England Workforce
PAR	participatory action research
DOC	Department of Correction
SWSC	study-wide steering committee
OJT	on the job training
